# Artificial Intelligence at the Intersection of Chemistry and Materials Science

**DOI:** 10.3390/ai7030089

**Published:** 2026-03-02

**Authors:** Tomas Gregan, Juraj Gregan

**Affiliations:** 1 Institute of Materials Chemistry, Technische Universität Wien (TU Wien), Getreidemarkt 9/165, 1060 Vienna, Austria; 2 Institute of Microbial Genetics, Department of Agricultural Sciences, BOKU University Vienna, Campus Tulln, Konrad Lorenz Strasse 24, 3430 Tulln an der Donau, Austria

**Keywords:** metal–organic frameworks (MOFs), artificial intelligence (AI), chemistry, materials, carbon capture, drug delivery, large language models, machine learning

## Abstract

Research on metal–organic frameworks (MOFs) bridges the fields of chemistry and materials science. MOFs consist of metal ions linked together by long organic molecules. These materials are known for their high porosity and large surface area, with numerous applications ranging from storage of various gases to medical uses. Recent developments show that artificial intelligence (AI) is revolutionizing the discovery and design of MOFs. Despite these advancements in AI-driven approaches in MOFs, many challenges remain in processes such as data quality assurance and experimental validation. In this perspective, we highlight recent progress in MOFs and discuss the role of AI in this truly interdisciplinary field.

## 1 Introduction

Artificial intelligence (AI) can be defined as the capacity of computers or other machines to exhibit or simulate intelligent behavior [1]. An important aim of AI is to design agents that can act in complex environments to achieve goals. Examples of AI techniques include machine learning, which enables systems to learn from data, neural networks, and deep learning [2]. Across many, if not all, scientific disciplines, AI is being used to integrate large datasets, refine measurements, guide experimental approaches, and generate reliable models integrated with scientific workflows for autonomous discovery [3]. In fact, these days it is difficult to discuss any topic in science without mentioning AI. Notable examples of how AI is actively contributing to various branches of science and education include the Gemini large language model winning a gold medal at the International Mathematical Olympiad 2025 [4]. Recently, DeepMind’s biology toolkit has been expanded with AlphaGenome, a tool that advances efforts to decipher the regulatory code of the genome. This deep learning model unifies multimodal prediction, long-sequence context and base-pair resolution into a single framework [5]. Although only a few AI-discovered or AI-designed drugs have reached clinical trials so far, AI is also accelerating drug discovery. A generative AI-discovered TNIK inhibitor for idiopathic pulmonary fibrosis showed promising results in a randomized phase 2a trial, representing a milestone for both pulmonary fibrosis therapy and AI-enabled drug discovery [6]. Deep learning-based virtual screening of over 1.4 billion compounds identified 82 candidates exhibiting antibacterial activity, illustrating a 90-fold improved hit rate over the screening experiment used for training [7]. Moreover, publishing scientific literature is no exception when it comes to leveraging AI. Scientific journals use AI tools such as iThenticate and Proofig to identify plagiarized text and figures as well as DataSeer’s natural language processing technology to produce reproducibility checklists [8]. However, such AI-generated reports should be carefully assessed by scientists or editors before any important decisions are made, as they may contain inaccuracies that require expert judgment to identify and correct.

Notably, in October 2024, two Nobel Prizes were awarded for work related to AI. John J. Hopfield and Geoffrey Hinton shared the Nobel Prize in Physics 2024 for their discoveries that enabled machine learning with artificial neural networks [9]. John Hopfield created an associative memory that can store and reconstruct images and other types of patterns in data. Geoffrey Hinton invented a method that can autonomously find properties in data and so perform tasks such as identifying specific elements in pictures [9]. The Nobel Prize in Chemistry 2024 was awarded to David Baker for computational protein design and the other half jointly to Demis Hassabis and John Jumper for protein structure prediction [10]. David Baker used computational protein design to create entirely new proteins. Demis Hassabis and John Jumper developed an AI model that can predict structures of proteins [10]. Although their pioneering discoveries were achieved without the aid of AI, in October 2025, the Nobel Prize in Chemistry was awarded to Susumu Kitagawa, Richard Robson and Omar M. Yaghi for the development of metal–organic frameworks (MOFs) [11].

These recent awards highlight the importance of MOFs and the use of AI in basic research as well as in commercial applications. In this perspective article, we briefly summarize the importance and limitations of using AI for studying MOFs, from the point of view of life scientists. We hope that this article will inspire not only AI specialists but also students and enthusiastic non-specialists to reach experts in chemistry, biology and materials science who study MOFs or other equally fascinating topics. By fostering such interdisciplinary interactions, we aim to spark new ideas and drive innovative research. Indeed, some of the most important and unexpected discoveries have been made at the interface of different scientific disciplines. Therefore, it is likely that the interdisciplinary research involving MOFs and AI will result in major breakthroughs pushing the frontiers of intelligent materials discovery.

## 2 MOFs—The Beginning and State of the Art

**Figure 1:**
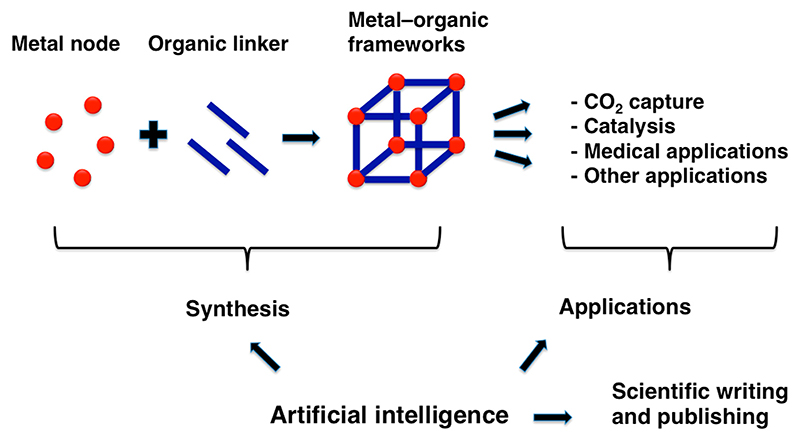
Artificial intelligence impacts many aspects of the metal–organic framework field.

MOFs are extended and ordered molecular structures that typically contain cavities and are built from metals and organic (carbon-based) molecules (Figure 1). MOFs represent a relatively new field in chemistry that is growing rapidly. One of the key persons in this field was Richard Robson, who developed a new class of materials, now called MOFs. In this material, he combined positively charged copper ions with a four-armed linker molecule that had a copper-binding group at the end of each arm [12,13]. Moreover, he was able to form a crystalline structure from these molecules with large cavities resembling sponge-like material. However, Robson’s initial constructs were relatively unstable and fragile. Only later did other scientists including Susumu Kitagawa and Omar Yaghi succeed in making MOFs that were stable, contained huge cavities and readily absorbed molecules such as methane and oxygen [14,15]. It is important to keep in mind the early days when the first MOFs were constructed because only then can one appreciate the tremendous progress in this field that we experience these days.

Currently, scientists are building on pioneering discoveries made by Richard Robson, Susumu Kitagawa and Omar Yaghi. Numerous types of MOFs have been produced using rational design so that the cavities have desirable properties. It is remarkable that because of their large internal surface area, only a few grams of MOFs with long linkers can have the surface area of a football field [16]. The surface area, pore size and chemical reactivity can be modulated by selecting the organic ligands and metal nodes within these MOFs. Such MOFs can act as catalysts, convert sunlight energy into chemical energy, capture carbon dioxide from open air or be used in medical applications [17–22]. This underscores that the importance of MOFs stems from their potential to tackle some of the challenges our world faces today, from climate change to healthcare.

One of the notable properties of MOFs is that many molecules that fill cavities do not bind to MOFs too strongly, so they can be relatively easily removed, making MOFs flexible and reusable. Many studies report static structures of MOFs but some of the most exciting studies describe the time-dependent phenomena that more accurately represent the real state of MOFs. Some MOFs can display framework flexibility while maintaining structural integrity throughout reversible framework changes caused by changes in the temperature, pressure, light or molecules residing in their pores. However, because detailed experimental information (e.g., atomic resolution and ultrafast phenomena) is difficult to obtain for such four-dimensional MOFs, their time-dependent behaviors remain far less explored than their steady-state properties [23,24].

While the first MOFs were made with a single linker, resulting in homogeneous pore environments, using multiple linkers with different connectivities now allows for access to new topologies. Such linkers introduce different functional groups into various pores of the same structure, demonstrating the excellent flexibility and tunability of MOFs [25,26]. Moreover, new types of MOFs can combine periodicity with aperiodicity. They contain an ordered lattice of connecting nodes and a network of aperiodically arranged linkers. Such structures can potentially be used to store information similarly to barcodes and QR codes [27].

If we want to translate scientific discoveries into business, we have to ask, how does this discovery solve a problem? MOFs have the potential to solve many problems that we are facing. Not surprisingly, there are a growing number of companies that aim to commercialize MOFs for harvesting water or capturing gases such as carbon dioxide. Through collaborations between academic research groups and commercial sectors, MOFs may deliver on their promise as game-changing materials across various fields. Creating platforms that integrate materials, process design, techno-economics and life-cycle assessment may help bring together chemists who design materials and engineers who focus on optimizing processes [28]. However, before MOFs can find their way into everyday use, several challenges such as high manufacturing costs have to be addressed [29,30]. AI can accelerate the synthesis of MOFs, while also opening the door to new materials that would otherwise not be discovered. While traditional methods often took years of experimental work, AI now makes discoveries possible in a much shorter time. This is particularly relevant to commercial sectors, as AI accelerates MOF discovery and application, reduces trial and error, and allows for precise fine-tuning of structures for maximum efficiency and scalability. AI-driven approaches, such as machine learning-based multi-objective frameworks that identify optimal MOF synthesis conditions while targeting specific structural properties [31], are likely to contribute to reducing the production costs of MOFs.

## 3 Using AI for Studying MOFs: Promises and Challenges

It is remarkable that more than 100.000 MOFs have been described in the literature [32]. The sheer number of available MOFs is overwhelming, yet our ability to identify the most suitable MOFs for specific applications, the ultimate goal for many researchers working in this field, remains limited [33]. This is one of the reasons why many scientists are turning to AI, hoping that it can help identify the most promising MOFs (Figure 1).

Traditional approaches to materials discovery rely heavily on stepwise synthetic chemistry, thermodynamic control and iterative experimental refinement. Although largely empirical in practice, these methods are firmly grounded in the principles of classical chemistry and solid-state physics. Optimizing material properties today clearly benefits from the incorporation of AI tools. Molecular simulations have long been a key tool in materials design, but recent efforts to combine molecular simulations with machine learning have accelerated the process [34,35]. By narrowing the list of MOF candidates for experimental synthesis, it directs researchers to the most promising MOFs, thus accelerating the MOF discovery process [36]. Simulating biomolecular dynamics with ab initio accuracy has long been a formidable challenge. To address this, an AI-based ab initio biomolecular dynamics system (AI^2^BMD) has been developed, capable of simulating full-atom large biomolecules, such as proteins, with close to ab initio accuracy [37]. Deep learning makes it possible to directly process text, image and graph representations of various materials, thus allowing for analysis of unstructured data and automated identification of features [38]. There are several examples where deep learning tools have been successfully applied for predicting MOF properties such as gas uptake and synthesizability [39,40]. Earlier studies applied computational methods such as density functional theory and grand canonical Monte Carlo simulations to perform high-throughput screening of various MOF properties. However, these approaches require substantial computational resources and extensive expert knowledge. Graph neural networks represent a rapidly expanding branch of machine learning models particularly well-suited for materials discovery [41]. Graph neural networks have been used to process molecular structural information. Several crystal graph neural networks have been applied to MOF performance prediction [42]. A novel multimodal graph neural network model named MMAGNN used the molecular graph structure of MOFs and the three-dimensional coordinates of atoms as inputs for predicting carbon dioxide adsorption in MOFs. The multimodal design reduced redundant information and feature interference, improving prediction accuracy and stability [43].

Using AI tools may unlock discoveries that have been so far out of reach. Recent break-throughs, including AlphaFold and diffusion-based models in inorganic crystal generation, show the enormous potential of AI tools in molecular structure-related discoveries [44,45]. It is not surprising that the Google DeepMind is trying to replicate the success of AlphaFold in predicting protein structure by applying AI to other scientific disciplines including materials science [46]. However, reducing the discussion of AI’s impact to generative methods would overlook how broad and deep the field really is. A recent *Nature* article on science perspectives for 2026 suggests that we will see growing numbers of AI tools that move beyond large language models. These approaches will emphasize small-scale AI models that learn from a limited pool of data and rather than producing text, process mathematical representations of information. Such small-scale AI models could potentially outcompete large language models in reasoning tasks [47,48]. There is a remarkable shift from conventional screening approaches to inverse generation driven by deep generative models. Inverse design of MOFs, which starts by targeting properties and works backwards to design desirable materials, is gaining importance [49]. AI agents based on reinforcement learning are able to learn by interacting with the environment. Reinforcement learning frameworks can successfully design MOFs that are important for the environmental application of direct air capture of CO_2_ [50]. It is also exciting that it is now possible for machines to discover a state-of-the-art reinforcement learning rule that outperforms manually designed rules [51].

**Figure 2:**
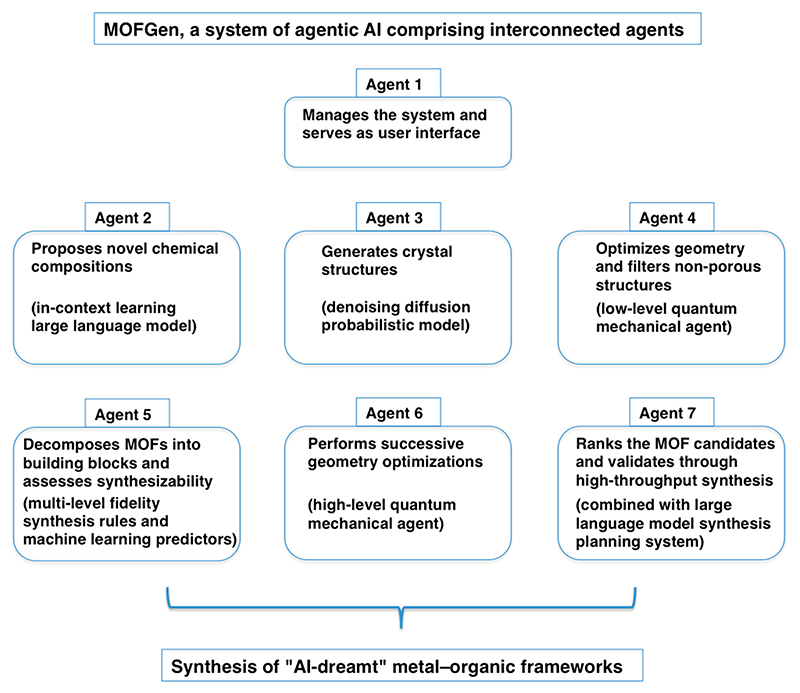
A simplified framework of MOFGen, illustrating how artificial intelligence (AI) can be used to design new metal–organic frameworks (MOFs) (adapted from Inizan et al. [53]).

AI tools provide enhancements for linking complex structures to targeted functions with unprecedented efficiency. The synergy between AI and our knowledge of MOFs has the power to revolutionize the way we design and produce materials [52]. One recent example of how to accelerate the discovery of MOFs is the system of an agentic AI called MOFGen, which generated hundreds of thousands of novel MOF structures [53]. Rather than relying on traditional chemical methods, which are often time-consuming and dependent on experimental heuristics and chemical intuition, MOFGen links specialized agents to facilitate the rapid exploration and discovery of synthesizable MOFs. Unlike traditional AI tools, which often rely on structured instructions and close supervision, an agentic AI is known for its ability to make sophisticated decisions in dynamic situations, enabling it to operate effectively in evolving environments with minimal human supervision [54]. MOFGen is a modular system composed of AI and computational agents, each defined as an entity that can act based on its environment to accelerate the discovery of novel MOFs (Figure 2). MOFGen combines large language models that propose novel MOF compositions, diffusion models that generate crystal structures, quantum mechanical agents that optimize and filter candidates, and synthetic-feasibility agents guided by expert rules and machine learning. By employing generative models and AI-driven methodologies, MOFGen accelerates the creation of “AI-dreamt” MOFs (Figure 2). The robustness of MOFGen was experimentally validated by synthesizing five “AI-dreamt” MOFs, whose crystal structures were in agreement with those generated by a diffusion model [53]. It is likely that in the future, we will see more sophisticated multimodal agents with an emphasis on deeper collaboration between agents and experimental methods.

Similarly to other research fields, frequently used applications of AI in MOF research include literature searches and data extraction to accelerate the discovery process [34]. Such AI tools transform how scientists engage with the literature, surpassing traditional approaches that rely on reading papers individually and manually collecting data. The recently introduced MOF-ChemUnity represents a literature-informed large language model for MOF research that links information derived from the literature to crystal structure and computational datasets [55]. The AI can accelerate the progress in the application of MOFs but only as long as high-quality data are findable and accessible. Using AI tools in combination with poor data quality may lead to misleading results. Therefore, it is important to ensure that published characteristics of MOFs are findable, accessible, interoperable, and reproducible (FAIR). Guidelines for the publication of experimental as well as computer-simulated data already exist (e.g., [56]). One of the barriers to improving AI applications in drug discovery is the lack of standardized, high-quality data [57]. A similar challenge is likely to hinder progress in materials discovery. Instead of relying on multiple, competing datasets, the field would benefit from a community-driven approach coordinated by an independent organization. High-quality structural data are particularly important for the success of AI tools. However, recent studies have uncovered significant error rates in MOF databases, posing a serious problem that hinders efficient, AI-driven discoveries. Therefore, it is important to regularly improve tools and algorithms that help to overcome this challenge (e.g., [58,59]). These tools can then be used to update curated sets of computation-ready MOF crystal structures designed for high-throughput computational materials discovery, such as the CoRE MOF database [60]. While AI offers significant opportunities for MOF research, its commercialization has also intensified concerns regarding the transparency and safety of AI models. To address these concerns, it is important to develop systems that rate AI models based on their completeness and openness for reproducibility, transparency, and usability [61]. There are growing concerns that AI agents may engage in deception or pursue goals that were not specified by human operators [62]. While researchers have warned of potential risks associated with AI, consensus on how to effectively address them remains limited [62]. Reinforcement learning agents that plan effectively over a long time horizon present particular risks [63]. Yoshua Bengio and his team are developing a system called Scientist AI that will act as a guardrail against AI agents. Scientist AI will predict the probability that an agent’s actions will lead to harm and, if required, block these actions [64]. No less important is the concern that using AI to produce or review MOF publications could compromise reliability and pollute the scholarly literature. Scientific journals now implement relevant measures, such as prohibiting reviewers from submitting manuscripts to large language models to generate reviews [8].

**Figure 3:**
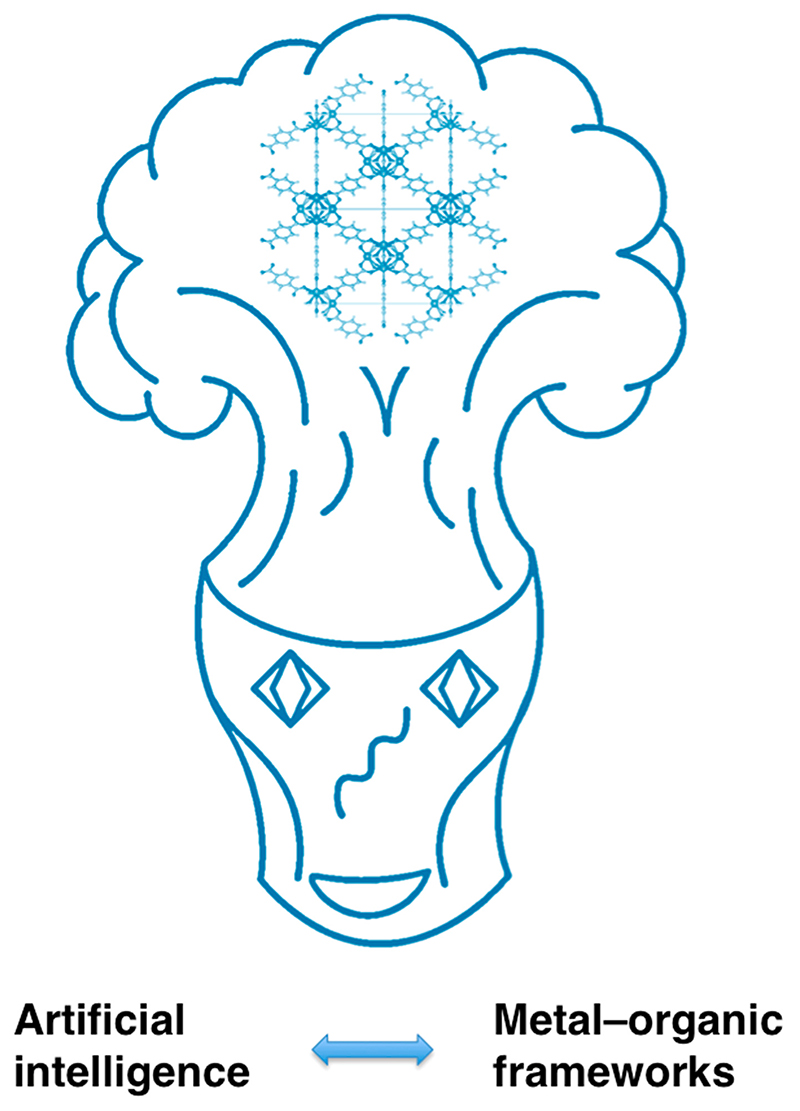
Conceptual illustration depicting the convergence of artificial intelligence (AI) with the field of metal–organic frameworks (MOFs). A machine-inspired, human-like head releasing a surreal, seemingly unbound cloud of ideas symbolizes the transformative potential of AI-driven MOF research. At the center of this abstract cloud stands a unit cell of MIL-125, an example of a highly porous and crystalline titanium (IV) dicarboxylate [70], depicting how AI utilizes data of known MOF structures to architect novel MOFs with reimagined, custom-designed structures and functionality. Please note that this is an abstract illustration using symbols rather than real images and does not accurately represent reality.

It is important to note that there are increasing concerns that current AI tools are not ideal for designing robust scientific questions that have the potential to push forward the frontiers of the field [65] and that they lack the curiosity and imagination needed for truly groundbreaking discoveries [66]. Therefore, efficient communication between experts in MOFs and AI specialists is of crucial importance. In this aspect, it will be important to strengthen user-friendly approaches for exploring available MOF data. This could be driven by chat-based analysis with natural language, similarly to the recently established CellWhisperer, which uses AI models to emulate data-centric conversations between biologists and informaticians [67], or ChatMOF, which predicts and generates materials with user-desired properties from natural language [68]. Because the functional characteristics of MOFs are complex and dynamic, the language-based representations easily interpretable by large language models are rather challenging. Therefore, it is important to develop further multimodal large language models for MOFs. One recent example is the L^2^M^3^OF, which integrates crystal representation learning with language understanding to process structural, textual and knowledge modalities [69]. We anticipate that natural language will become a useful channel for MOF data analysis and it will be an important part of AI-based research assistants. This also means that there will be a growing demand from MOF experts trained in chemistry for integration of AI tools and robotics. This transition is essential for this field to stay competitive with other fields of science. This is not just an updating of the traditional methods. This is a dramatic change, with critical implications for the entire field of MOFs and far beyond (Figure 3) [52].

Taken together, research in the field of MOFs is a good example of how AI can transform traditional scientific approaches and accelerate new discoveries. AI is extremely efficient in analyzing large data and drawing conclusions about MOFs. We expect that AI agents that integrate several large language models to carry out multi-step processes with little human oversight will be extensively used in MOF research. It is also possible that predictive AI that involves statistical analysis and machine learning will be used to predict potential future outcomes. Furthermore, quantum computers could enhance the impact of AI on chemistry and materials science. However, extensive use could also expose more errors that AI agents are prone to. AI safety is a hotly debated topic and emerging AI-related laws and regulations may also affect the use of AI tools in materials discovery. It is also becoming increasingly clear that AI will disrupt the job market, leading to unusually severe disruptions. However, in our opinion, science is not just a pipeline that converts data into conclusions. For tasks that require thorough conceptual reasoning, understanding of abstract ideas and scientific judgment, we still need experts who understand both the strengths and limitations of results obtained by traditional (pre-AI) approaches. In the era of AI, we should not forget the techniques and approaches that may seem old-fashioned but have stood the test of time. Thus, it is evident that, while AI can be considered a valuable collaborator, it currently lacks the capacity to fully substitute a conventional scientist. Only the synergy resulting from combining AI with our knowledge of previous achievements in MOFs has the power to revolutionize the way we design and produce materials.

## Data Availability

Not applicable.

## Abbreviations

The following abbreviations are used in this manuscript:

AI: Artificial intelligence
MOF: Metal–organic framework
